# The Uses of Mileage Recorders and Speedometers

**Published:** 1910-01-08

**Authors:** 


### Motoring Notes.

### THE USES OF MILEAGE RECORDERS AND SPEEDOMETERS.

Generally one of the first things that the average owner thinks of buying, after he has received his car with its equipment of tools and lamps, is a speedometer, with a mileage recorder combined. While either of these instruments can be bought separately, there are many who consider that a combination of the two in one instrument is better. In the first place only one drive is required for the combination instrument; in the second, one instrument acts as a check on the other, while the accuracy of the modern mileage recorder is beyond suspicion within very trifling limits. Yet the fact remains that flexible shafts will break, and may go unnoticed in the case of the distance recorder, since the figures change so slowly, whereas if it is combined with the speedometer the speed indicator will return to zero and the failure will at once be noticed. On the other hand if the accuracy of the speedometer is doubted it is an easy matter to run the car over a given distance, keeping the speed as recorded by the speedometer as uniform as possible, and, by timing the car over this distance test the accuracy of this portion of the instrument. If this is done for about a mile each at, say, ten, twenty, and thirty miles per hour, the results should prove a very good check on the accuracy of the instrument.

### The Odometer.

Many owners do not realise how useful the odometer, or distance recorder, really is, and only use it to secure a record of their mileage for the season. However, almost everyone wants to know how satisfactory his car is from an economic standpoint, and this should be judged by the expense per mile rather than per day or per month. It will thus be readily seen that the odometer is a very important instrument, and its use should be thoroughly understood. Probably the greatest expense in the upkeep of a car is the item of tyres, the economy of any certain make depending not solely on the first cost, but also on the mileage it gives. It is a very simple matter to keep account of tyre mileage by means of the distance recorder. It is only necessary to put down the reading in one's diary when the tyre is first fitted, and then, when it is removed from the rim for replacement or re-treading, to take another reading. It might here be said that all motorists should keep a diary, if only a simple one of the vest-pocket size. At first one can start with simply keeping each day's mileage. It will be found rather interesting if the destination is also recorded, and very often the distance to and from certain places may prove of interest. If the mileage recorder is provided with a trip dial, its indications may be recorded directly, otherwise the mileage shown on the season dial may be recorded at the end of each day's run. In case of a puncture, the wheel on which it occurs, together with the mileage recorded at the time of the puncture, should be recorded.

There are several things that should be done to a car every now and then. For instance, the differential should be cleaned out and relubricated every 1,000 miles. When this is done a record should be made in the diary, and this can be referred to to see when it is necessary to repeat the process. Most of the petrol used is obtained at the local garage, and its amount can be ascertained from the monthly bill; but in case of tours the quantity of petrol bought whilst away from home may be recorded, as well as the price paid. A record of the oil consumption can be kept in a similar manner. Another use of the odometer which seems to be almost unknown to the average motorist is as an aid to finding one's way from place to place either by means of a road book or by a map. Let us say that we find after passing through the centre of a given town we should take a road to the right 2½ miles from this centre. On passing through the centre we find that the mileage recorder reads, say, 24.7 miles, then at about 27 miles it is time to commence to look for our road.

### The Speedometer.

Turning now to the uses of the speedometer, perhaps the principal one which occurs to the average owner when the subject is mentioned is either to enable the driver of the car to keep within the legal limit or to see how fast the car can go, the reply depending on the temperament of the individual. As a matter of fact, it would hardly pay to go to the expense of fitting a speedometer for these reasons alone. For instance, one can easily find the extreme speed of a car by timing it over a given short stretch and figuring out the speed. Probably one of the greater missions of the speedometer is to teach the average motorist the folly of wanting a car that will do everything on the top speed. It is also excellent for determining the best time for changing gears. Let us take, for instance, a car equipped with four speeds. It is probable that, on what we may call a normal engine speed, the car will be capable of about six miles on the first, fifteen on the second, up to thirty on the third, and from thirty upwards on the top, according to the power of the engine. However, the car may be capable of being driven down to less than ten miles an hour on the top speed and of surmounting ordinary hills on this gear. It will probably also be found that, if a long hill is encountered, the speed may fall to fifteen miles per hour on the top gear, but that by changing down to the third at the proper time a tyenty-mile rate can be easily maintained all the way up the hill. No exact rules can be laid down as to the most advantageous time to change gears on a hill, as the gradient, the characteristics of the car and motor are great factors in the problem, but to the novice the speedometer should be a great help in this matter. From the foregoing it will be seen that as a means of checking our running cost the distance recorder is practically indispensable and the speedometer most helpful in driving, especially to the beginner.

